# Analysis of carotid vulnerable plaque MRI high-risk features and clinical risk factors associated with concomitant acute cerebral infarction

**DOI:** 10.1186/s12872-023-03199-7

**Published:** 2023-03-30

**Authors:** Yongxiang Tang, Jinping Zhang, Weizhou Liu, Wei Jin, Shijian Li, Zhen Qian, Xiaoquan Kong, Ran Zhang, Juanjuan HU, Baolin LI, Weiming Yuan, Yifan Zhang

**Affiliations:** 1 Department of Medical Image Center, Tongling People’s Hospital, Bijiashan Road 468, Tongling, 244000 Anhui China; 2 Department of Neurology, Tongling People’s Hospital, Bijiashan Road 468, Tongling, 244000 Anhui China

**Keywords:** Carotid vulnerable plaque, Intraplaque hemorrhage, Necrotic lipid core, Ulcer, Magnetic resonance imaging, Acute cerebral infarction

## Abstract

**Background:**

This study aimed to investigate the correlation between the high-risk characteristics of high-resolution MRI carotid vulnerable plaques and the clinical risk factors and concomitant acute cerebral infarction (ACI).

**Methods:**

Forty-five patients diagnosed with a single vulnerable carotid plaque by MRI were divided into two groups based on whether they had ipsilateral ACI. The clinical risk factors and the observation values or frequency of occurrence of high-risk MRI phenotypes of plaque volume, LRNC, IPH and ulcer were statistically compared between the two groups.

**Results:**

A total of 45 vulnerable carotid artery plaques were found in 45 patients, 23 patients with ACI and 22 patients without ACI. There were no significant differences in age, sex, smoking, serum TC, TG and LDL between the two groups (all P > 0.05), but the ACI group had significantly more patients with hypertension (P < 0.05) and the without ACI group coronary heart disease (P < 0.05). The volume of vulnerable carotid plaque in the group with ACI (1004.19 ± 663.57 mm3) was significantly larger than that in the group without ACI (487.21 ± 238.64 mm3) (P < 0.05). The phenotype of vulnerable carotid artery plaque was 13 cases of LRNC, 8 cases of LRNC + IPH, 5 cases of LRNC + Ulcer, and 19 cases of LRNC + IPH + Ulcer. There was no significant difference in this distribution between the two groups (all P > 0.05) with the exception of LRNC + IPH + Ulcer. The 14 cases of LRNC + IPH + LRNC + IPH + Ulcer (60.87%) in the group with ACI and was significantly greater than the 5 (22.73%) in patients without ACI (P < 0.05).

**Conclusion:**

It is preliminarily thought that hypertension is the main clinical risk factor for vulnerable carotid plaques with ACI and the combination of plaque volume with vulnerable carotid plaque and LRNC + IPH + Ulcer is a high-risk factor for complicated ACI. It has high clinical therapeutic value due to the accurate diagnosis of responsible vessels and plaques with high-resolution MRI.

## Introduction

Stroke has become the leading cause of death in our country and is one of the most important causes of death and disability in the modern society. A central nervous system vascular event, acute cerebral infarction (ACI) is the most common type of stroke, 69.6–70.8% of stroke [1]. Vulnerable carotid plaque is an important factor inducing ACI, causing 15-30% of them [2]. Therefore, timely and accurate discovery and evaluation of vulnerable carotid plaques are of great significance for accurate diagnosis of responsible vessels and plaques accompanied by ACI, and understanding them will provide an important basis for the selection of clinical plaque treatment methods.Previous imaging examinations were based on the measurement of carotid lumen stenosis and plaque surface irregularity to determine whether carotid plaque would induce plaque shedding and lead to ACI, but with certain limitations [3]. Current research suggests [4, 5] that radiographic interpretation of vulnerable plaques, large lipid core hemorrhage (large necrotic lipid core, LRNC), plaque intraplaque hemorrhage(plaque intraplaque hemorrhage, IPH), surface ulcer (Ulcer), and other high-risk characteristic factors are important. These factors can induce plaque shedding, leading to concomitant ACI. There have been future predictive assessments of cerebrovascular events within 2 weeks after the discovery of vulnerable carotid artery plaques, but the occurrence of finding concomitant ACI after immediately plaque examinations is rarely reported [2, 3]. In this study, high-resolution carotid artery wall MRI was adopted to evaluate vulnerable carotid plaques, which is a simple, rapid and risk-free diagnostic method. It can not only evaluate plaque size and shape but also accurately diagnose high-risk factors among plaque components. The purpose of this study was to explore the correlation between the high-risk characteristics and clinical risk factors for carotid artery vulnerable plaque analyzed by high-resolution MRI and ACI at the time of examination to provide more clinical information for decision-making.

## Materials and methods

### General data

All patients were inpatients from Tongling People’s Hospital were eligible for inclusion if (1) ACI was clinically suspected or had been confirmed by imaging and (2) there was only one atherosclerotic plaque located within 3 cm above or below the bifurcation of the first lateral common carotid artery and internal carotid artery. To ensure as far as possible that the observed single vulnerable carotid plaque was the only plaque associated with ACI liability and the examination was safe, patients with the following conditions were excluded from this study: (1) thrombus in the distal artery or its branches of the plaque was determined by head and neck magnetic resonance angiography (MRA) or conventional MRI; (2) the patient had atrial fibrillation or cerebral hemorrhage; (3) the patient had a history of radiotherapy to the neck; and (4) there were contraindications on magnetic resonance imaging. Carotid artery high-resolution MRI vascular wall imaging was completed after admission. The majority of patients with undiagnosed ACI underwent simultaneous craniocerebral diffusion-weighted imaging (DWI) examination, while a small number of patients underwent simultaneous craniocerebral MRI or MRA examination. The possible clinical risk factors of all patients, including age, sex, hypertension, diabetes, coronary heart disease, smoking history, blood lipid levels and other data, were obtained from the inpatient records. All included patients were informed of the purpose of the examination and signed the examination informed consent.

A Philips Achieva 3.0-T magnetic resonance scanner dedicated 8-channel carotid wall imaging phased array coil (Bioengineering Laboratory of Tsinghua University). First, 2D-TOF MRA imaging was used to scan the neck and obtain the general MRA map of the neck blood vessels. A high-resolution carotid sequence was located. The scanning center was placed at the bifurcation of the carotid artery, and the patient was instructed to relax during the scanning process, breathe calmly and minimize swallowing. Carotid artery TOF, T1WI, T2WI and simultaneous noncontrast angiography and intraplaque hemorrhage(SNAP) images were obtained, whlie conventional brain MR images were taken using T1WI, T2WI, T2 FLAIR and DWI sequences. The specific parameters of the main imaging sequences are listed in Table 1.

**Table 1:** Carotid MR vessel wall and cranial imaging parameters

	Carotid plaque				Cranial
	T1WI	T2WI	TOF	SNAP	DWI
**TR (ms)**	800	4800	20	9.8	2724
**TE (ms)**	10	50	5	4.7	86
**Flip angle**	90°	90°	20°	α = 5° θ = 11°	90°
**FOV (mm** ^**2**^ **)**	160 × 160	160 × 160	160 × 160	160 × 160	160*160
**Resolution**	0.6 × 0.6	0.6 × 0.6	0.6 × 0.6	0.35 × 0.35	-
**Layer thickness (mm)**	2	2	2	0.6	5

### Image analysis and processing

#### Interpretation of plaque

All examination images were transmitted to a Philips Nebula 6.0.2 postprocessing workstation. The carotid artery bifurcated layer was used as a reference, and the location and morphology of the narrowest lumen layer and plaque contents were used for multidirectional and multisequence imaging evaluation and measurement of carotid artery plaques. Images well matched between sequence and multicontrast sequence images were selected, and images well matched between SNAP sequence and multicontrast sequence images were selected for study. A senior attending physician and deputy chief physician made the imaging diagnoses. The site and size of the plaque were recorded in detail, and MRI characteristics such as IPH, LRNC and ulcer were interpreted. The reading criteria were based on previous literature [5]. See in Table 2.

**Table 2:** Signal performance of carotid atherosclerotic plaque components on each serially weighted image

	T1WI	T2WI	TOF	SNAP
**IPH**	Hypersignal	Hyposignal/Hypersignall	Hypersignal	Hypersignal
**LRNC**	Isosignal/Slightly hypersignal	Hyposignal	Isosignal	Slightly hyposignal
**Ulcer**	Surface irregularity	Surface irregularity	Surface irregularity	Surface irregularity

Note: The signal level of each sequence of images is judged with reference to that of the sternocleidomastoid muscle at the same level

#### Diagnosis of ACI

The criteria for the diagnosis of ACI were as follows: (1) The cerebral parenchyma on cranial DWI showed patchy hypersignals of varying sizes, and the signs and symptoms of acute stroke were clinically suspected to be ACI. (2) In the routine plain scan of the brain, the brain parenchyma showed abnormal flake lesion of different sizes, equal T1 or slightly longer T1 and equal T2 or slightly longer T2 signals, and T2 FLAIR showed a slightly higher homogeneous signal, or MRA showed no obvious lumen stenosis in the corresponding supplying artery branch of the distal segment of the plaque, and there were clinical signs and symptoms of acute stroke that roused suspicion of ACI.

### Statistical analysis

SPSS 26.0 statistical software was used for analysis. Measurement data conforming to a normal distribution are presented as mean ± standard deviation and were compared between the two groups by the independent-sample t test. Measurement data not conforming to a normal distribution are presented as median and interquartile spread and were compared between the two groups by the Mann‒Whitney U test. Count data are presented as number (%) and were compared between the two groups by the standard four-cell-table chi-square test. P < 0.05 was statistically significant.

## Results

### Clinical data

The 45 cases included 30 males and 15 females. Their age ranged from 37 to 90 years old, with an average age of 70.13 ± 10.33 years old. A total of 45 plaques were found in patients with vulnerable carotid artery plaques, who were divided into two groups: 23 patients with ipsilateral ACI and 22 patients without ipsilateral ACI. Comparison of general clinical risk factors between the two groups showed that the ACI group was more likely to have hypertension and less likely to have coronary heart disease (both P < 0.05), while there was no significant difference in other factors (all P > 0.05). See Table 3 for details.

**Table 3:** Comparative analysis of risk factors among the clinical data of the two groups

Clinical risk factors	Group		P
	**Without ACI (*n*** **= 22).**	**ACI (*n*** **= 23)**	
General clinical data			
Age (year)	71.93 ± 7.87	75.73 ± 6.50	0.096$^\blacktriangle$
Sex (male)	12(54.6)	18(78.3)	0.092$^\blacktriangle$
Diabetes (n (%))	7(31.82)	4(17.39)	0.260$^\blacktriangle$
Hypertension (n (%))	10(45.6)	22(95.7)	0.000*
Coronary heart disease (n (%))	13(59.1)	4(17.4)	0.004*
Smoking (n (%))	3(13.6)	5(21.7)	0.477$^\blacktriangle$
Laboratory tests			
High-density lipoprotein (mg/dl)	1.05 ± 0.30	1.09 ± 0.32	0.796$^\blacktriangle$
Low-density lipoprotein (mg/dl)	2.71 ± 1.36	2.42 ± 0. 82	0.581$^\blacktriangle$
Total cholesterol (mg/dl)	4.55 ± 1. 61	4.03 ± 0.99	0.391$^\blacktriangle$
Triglycerides (mg/dl)	1.76 ± 0. 71	1.57 ± 0. 88	0.217$^\blacktriangle$

Note: $$^\blacktriangle$$P > 0.05; *P < 0.05

### MRI analysis

**Fig. 1:**
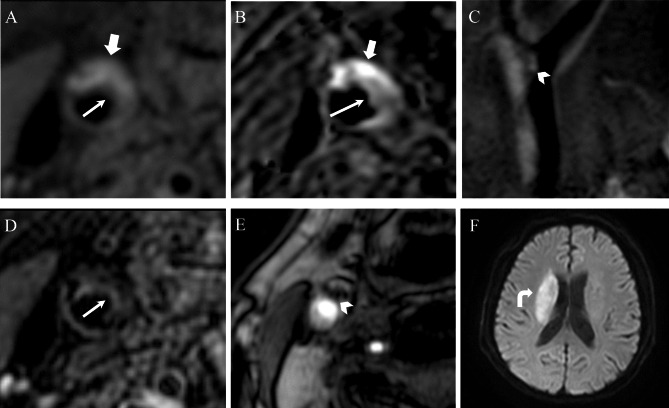
Patient, male, 70 years old. High resolution wall imaging of the internal carotid artery diagnosed vulnerable plaques in the right internal carotid artery with accompanying ipsilateral acute cerebral infarction. The following MRI images were obtained: (**A**) Axial T1WI clearly showed the plaque LRNC with hypersignal (fine arrow) and IPH with hypersignal (thick arrow);(**B**) Axial SNAP clearly showed that patch LRNC showed slightly hyposignal (thin arrow) and patch IPH showed significantly hypersignal (thick arrow);(**C**) Sagittal T1WI clearly showed a Ulcer with an irregular surface mixed signal (arrowhead);(**D**) Axial T2WI clearly showed hyposignal of plaque LRNC (thin arrow);(**E**) Axial 2D-TOF MRA raw image showed a patch of irregular surface Ulcer hyposignal (arrowhead);(**F**)Craniocerebral axial DWI showed ACI with flaky hypersignal surrounding the right lateral ventricularl (curved arrow)

The vulnerable carotid plaque volume was significantly larger in the group with ACI (1004.19 ± 663.57 mm^3^) than in the group without ACI (487.21 ± 238.64 mm^3^) (P < 0.05). The phenotype of vulnerable carotid artery plaque was distributed as 13 cases of LRNC, 8 cases of LRNC + IPH, 5 cases of LRNC + Ulcer and 19 cases of LRNC + IPH + Ulcer (Fig. 1). LRNC + IPH + Ulcer was significantly more common in the group with ACI (P < 0.05), but the others had similar rates. See Table 4 for details.

**Table 4:** Comparative analysis of high-risk factors for vulnerable carotid artery plaques between the two groups

Carotid artery vulnerable plaque high-risk factor	Group		*p*
	**Without ACI (*n*** **= 22).**	**ACI(*n*** **= 23)**	
Plaque volume (mm^3^)	487.21 ± 238.64	1004.19 ± 663.57	0.005*
LRNC (n (%))	10(45.45)	3(13.04)	0.016*
LRNC + IPH (n (%))	3(13.64)	5(21.74)	0.477$^\blacktriangle$
LRNC + Ulcer (n (%))	4(18.18)	1(4.35)	0.140$^\blacktriangle$
LRNC + IPH + Ulcer (n (%))	5(22.73)	14(60.87)	0.010*

Note: $$^\blacktriangle$$P > 0.05; *P < 0.05

## Discussion

From the analysis of general clinical data of risk factors for carotid plaque formation, risk factors for carotid atherosclerosis formation include age, sex, hypertension, diabetes, coronary heart disease, smoking history, lipid level, etc. [6]. This study found that there was no significant difference in age, sex, diabetes, smoking history, or lipid levels between the groups with and without ACI at the time of examination of vulnerable plaques, indicating that these factors are risk factors for the formation of atherosclerotic vulnerable plaque but are not enough to be main indicators for determining whether there is ACI at the time of examination. There are significant differences in factors of hypertension and coronary heart disease. Patients with hypertension are more likely to have ACI. The main reason may be that patients with hypertension can experience hemodynamic changes, and the impact of the inner wall of blood vessels is stronger than that in patients without hypertension, which easily leads to the rupture of vulnerable plaques. The severity of coronary heart disease is positively correlated with the number of vulnerable carotid plaques [7], but no correlation has been reported between coronary heart disease and ACI. The obvious increase in coronary heart disease without ACI in our cohort is not explained, but further research in larger samples may suggest an explanation.

  Although obvious progress has been made in the treatment of ACI in recent years, such as intravenous thrombolysis and mechanical thrombectomy, most patients are still left with serious disability or even die [6]. Therefore, the key to reducing the burden of stroke lies in effective stroke prevention. Rupture and detachment of vulnerable carotid plaques is one of the main factors leading to ACI. Therefore, it is particularly important to predict ACI through accurate diagnosis of vulnerable carotid plaques. However, accurate diagnosis of the responsible vessels and plaques of ACI is also very important, as it can greatly help in choosing the best clinical treatment. This study tries to make some contribution to this decision-making.

  Compared with other traditional imaging examinations, high-resolution MRI has become the best technique for the noninvasive evaluation of vulnerable carotid plaques due to its excellent soft tissue imaging and multidirectional reconstruction. Because the carotid artery is an elastic artery with well-developed smooth muscle and strong vascular compensation remodeling, carotid lumen stenosis cannot be used as an independent risk factor for severity determination. Plaques with mild to moderate stenosis may still lead to ACI, indicating that lumen narrowing is not the only predictor of cerebrovascular events [8]. In recent years, it has been considered that the determination of plaque stability is more concerning and is very important for the occurrence and prediction of ACI, having become a research hotspot. Atherosclerotic plaque stability is determined by accurate analysis of plaque composition, blood supply and other factors. Plaque volume, LRNC, IPH and surface Ulcer have been identified as high-risk factors for vulnerable plaque [9–13]. In this study, we made comparisons of single factor and multiple factors among the high-risk characteristic factors diagnosed on MRI as vulnerable plaques to understand the degree of their contribution to concomitant ACI, which has not been found in the research literature and is worth further discussion.

  In this study, the volume of carotid artery vulnerable plaques was correlated with the frequency of ACI at the time of examination. The plaque volume in the ACI group was significantly larger than that in the non-ACI group. This finding is consistent with previous studies that extended the follow-up time after MRI diagnosis of vulnerable plaque to track the incidence of secondary ipsilateral ACI [14, 15]. The reason this happens may be that the ruptured plaque that caused the ACI arose from a vulnerable plaque that had not ruptured. A combination of factors, such as bleeding inside the plaque, aggravation of the inflammatory reaction, and formation of adhesion thrombosis caused by ulcer, led to a significant increase in plaque volume.

  The study also found that a high-risk factor was present for a common group of vulnerable plaque -- LRNC, LRNC + IPH, LRNC + ulcer and LRNC + IPH + ulcer. There was no significant difference between LRNC + IPH and LRNC + ulcer in frequency of occurrence in the ACI group (all P > 0.05). It may be that a plaque ruptured after bleeding on the basis of LRNC but has not yet formed a visible superficial ulcer or a plaque with a thick and intact fibrous cap despite IPH. As a result, LRNC + IPH vulnerable plaques have similar chances to arise with or without ACI. A remnant surface of a plaque ruptured by a thin fibrous cap on an LRNC or a surface accompanied by a plaque LRNC is old and has not yet ruptured. There is a similar chance of an LRNC + ulce vulnerable plaque with or without ACI. However, when LRNC + IPH + Ulcer high-risk factors were present, there was a significant difference (P < 0.05) in the incidence of ACI. This may be because vulnerable plaque bleeding on top of the LRNC and ruptured a thinner fibrous cap caused a superficial clot to form in the affected vascular cavity. ACI was associated with thrombus detachment. Therefore, if LRNC, IPH and ulcer, three high-risk factors, are all present, they make an accurate diagnosis of a responsible vessel and a responsible plaque substantially more likely with ACI, providing important evidence for targeted clinical treatment. It is worth noting that in this group, the incidence of LRNC alone increased significantly in the group without ACI, which may be because there were more cases of LRNC alone accompanied by a relatively thick fiber cap and temporarily unruptured vulnerable plaque in this group.

  This study has the following limitations: (1) We did not follow up patients diagnosed with vulnerable carotid plaque for future ACI development. (2) The sample size was small, so larger studies are needed in the future.

  In conclusion, hypertension is preliminarily considered to be the main clinical risk factor for vulnerable carotid plaques in patients with ACI. Larger vulnerable carotid artery plaques and the combination of LRNC + IPH + ulcer are high-risk factors for accompanying ACI. Therefore, they are of high value for making an accurate diagnosis of the responsible vessels and plaques in the presence of ipsilateral ACI at the time of examination and are therefore important for guiding clinical follow-up treatment.

## Data Availability

The datasets used and/or analyzed during the current study are available from the corresponding author on reasonable request.

## Abbreviations

ACI: Acute cerebral infarction
MRI: Magnetic resonance imaging
IPH: Intraplaque hemorrhage
LRNC: Lipid-rich necrotic core
SNAP: Simultaneous non-contrast angiography and intraplaque hemorrhage
TOF: Time-of-flight
